# 2020 Vision of Frontiers in Striated Muscle Physiology

**DOI:** 10.3389/fphys.2020.00292

**Published:** 2020-04-17

**Authors:** Peter J. Reiser, Paul M. L. Janssen

**Affiliations:** ^1^Division of Biosciences, College of Dentistry, The Ohio State University, Columbus, OH, United States; ^2^Department of Physiology and Cell Biology, College of Medicine, The Ohio State University, Columbus, OH, United States

**Keywords:** striated muscle, future, skeletal, cardiac, physiology

*Frontiers in Striated Muscle Physiology* has published just over 400 articles, all online and freely accessible to everyone, since its inception in 2010. Of particular note is the fact that more than 75% of the published articles in this Journal have been submitted as contributions to Research Topics with clearly defined and unique themes. What is striking is that, with a primary focus on striated muscle physiology, a vast majority (two-thirds) of the 32 Research Topics to-date have included a focus, not necessarily singular, on skeletal or cardiac muscle disease, pathology or pathophysiology. Three other areas that have been well-represented by Research Topics are aging, muscle wasting and fatigue/weakness. The remaining 25% of Research Topics had a primary focus on fundamental properties of striated muscle. Therefore, this Journal has been, and likely will remain, an important venue for the dissemination of a substantial amount of cutting-edge knowledge to better understand not only fundamental striated muscle physiology but also conditions that lead to significant decrements in muscle function. All of this success stems from the hard work of the authors and the associate editors, guest editors, and review editors who collectively represent a broad community of international scientists with an interest in open-access publishing.

Looking ahead for what are likely trends in the field of striated muscle physiology, several areas standout based upon what has evolved over the past decade. There has been a clear surge of studies that were focused on sex differences in virtually all physiology systems, including skeletal muscle and the heart, over the past 10 years and many published reports have revealed sex-related differences in the human population, as well as in model organisms. The results of a PubMed search with “muscle sex difference” as a search term, indicates a peak in the number of results in 2009, followed by a 3-year period with a small decrease in the number of annual publications. Beginning in 2013 there was a clear jump in the number of results, with a fairly steady increase through 2018 (the indicated number was lower for 2019 when the search was conducted and that might have been due to an incomplete compilation of results at that time). The increase in the number of published articles on sex-related differences coincides with an awareness of the importance of the inclusion of both sexes in the design and execution of studies of all physiological systems.

A more complete understanding of mechanisms that contribute to the maintenance of, or decrements in, muscle mass in association with aging, myopathies, and other diseases, such as cancer and AIDS, has been a goal for several decades and it is imperative that this remain a major focus well into the future. Not only is a loss of muscle mass and strength a major concern for many individuals, the physiological changes in muscle with reduced mass and the impact on recovery from muscle injury are not well-understood. A major goal should be understanding how muscle mass can be maintained during normal aging and in the context of specific myopathies and, more generally, in association with a broad spectrum of diseases.

It is well-recognized that skeletal muscle and cardiac muscle have the potential to express a myriad of sarcomeric protein isoforms that change during the course of normal development, in association with disease and following skeletal muscle injury and myocardial infarction. Given the large number of genes that code sarcomeric proteins that are expressed in striated muscle and the extensive alternative splicing that is known to occur in striated muscle, the number of possible combinations of expressed isoforms and, therefore, of the potential number of fiber types, is extraordinary. Yet it is also well-recognized that there is a limited number of fiber types that actually exist in striated muscles. How coordination of gene expression is executed to yield a very complex but finite number of combinations of sarcomeric protein isoforms, that directly regulate contractile properties of skeletal and cardiac muscle, is not well-understood. Elucidating the mechanisms that coordinate sarcomeric protein expression, given the impact on striated muscle function, should remain a major focus of investigation.

The study of the impacts of environmental changes, such as global warming and a greater prevalence of air pollution in cities, on many physiological systems, especially cardiovascular, and respiratory, has been intensely studied. However, with the likely persistence and worsening of these changes, rigorous investigations of the impact on not only the human population, but on all organisms, must remain as an elevated priority.

One area of investigation that is clearly on the rise is skeletal muscle exosomes—released extracellular vesicles. A PubMed search for “muscle exosomes” generated fewer than 10 hits annually, prior to 2013. Since that year, the annual number has grown steadily, reaching 127 in 2018. This is an area that is expected to grow substantially as more is learned about the contents of muscle exosomes, their targets and roles in pathophysiology. A review of skeletal muscle exosomes, with a focus on their involvement in metabolic diseases, was recently published in this Journal by Rome et al. (2019). The authors identified several areas in which more studies are needed to better understand the role of muscle exosomes in health ad disease. A very similar surge is found in a PubMed search for “cardiac exosomes.” More than half of all the hits in PubMed have been published in just the last 2 years. Combined, striated muscle exosomes point to a rapidly developing field that is indicating a role that striated muscle plays in the possible regulation and tuning of other systems in the body.

An additional area in which substantial advancement is anticipated in the near future is the utilization of high resolution cryo-electron microscopy. Given the inherent highly organized intracellular structure of striated muscle, the three-dimensional relationships of organelles and proteins to each other is critically important for proper function. Cryo-EM has the potential to yield an understanding of the precise structural relationships among intracellular components with near atomic resolution. There is a sense that exciting discoveries are inevitable with this imaging modality.

Molecular dynamics is also an emerging tool that is seeing a rapid increase of use in the striated muscle field. The computational analysis of each atom within a protein over time allows the study of protein interactions at the very single molecule level. Although currently limited to small time domains, and to the interaction of only a few proteins, with increased computational speed, eventually more complex systems, such as the sarcomere, can be modeled. With further enhancements in computational power, we should eventually be able to go through a contraction cycle, allowing us to learn the molecular nuances that make this intriguing sarcomere structure function. In addition, this will allow us, likely in combination with cryo-EM, to discern the impact of single amino acid mutations.

Rapid growth is also seen in the study of post-translational modifications (PTMs). This is of particular interest for proteins involved in the contractile aspects of striated muscle, as the vast majority of muscles consists of sarcomeric proteins that are stoichiometrically expressed. Thus, regulation of function does not necessarily take place via the amount of proteins expressed, unlike processes such as ion channels, and cell signaling that are predominantly regulated by the amount of protein. Since the contractile proteins are expressed at a fixed ratio, the majority, if not all, of the regulation of the function of these proteins relies on post-translational modifications. A challenge is to capture these modifications in their dynamic state, as several of these PTMs are labile, and can rapidly occur or dissipate. For instance, beta-adrenergic stimulation results in key phosphorylations in several proteins involved in contractile regulation, including phospholamban and troponin-I. This phosphorylation process occurs in mere seconds, or even faster, and constitutively active phosphatases can de-phosphorylate also in a very short time frame. Reliably capturing and analyzing the levels of PTMs will therefore be a challenge, but when achieved likely will yield a high pay-off in providing critical data on regulation of striated muscle function.

The past 10 years has been a period of great success for this Journal, in terms of the quality and scope of articles that have been published. Since 2011, the Journal has been growing on average 37% per year, with nearly 550 submissions to-date, and a 60% growth in manuscript submissions in the last 6 months, compared to the preceding 6-month period. Much of this success stems from the engagement of authors in response to *Research Topics*. Suggestions for future topics are welcome from all readers and may be submitted to either of the authors of this Perspective for initial consideration and potential development.

## Author Contributions

PR and PJ wrote the manuscript.

## Conflict of Interest

The authors declare that the research was conducted in the absence of any commercial or financial relationships that could be construed as a potential conflict of interest.
